# The Neglected Care Burden and Unmet Needs of Informal Caregivers of Stroke Survivors. A Qualitative Study

**DOI:** 10.1111/scs.70294

**Published:** 2026-07-06

**Authors:** Linn Wagner Sønderby, Maria Søe Mattson, Jacob Liljehult, Monique Mesot Liljehult, Dorthe Overgaard, Signe Stelling Risom

**Affiliations:** ^1^ Department of Nursing and Nutrition University College Copenhagen Copenhagen Denmark; ^2^ Department of Neurology Nordsjællands Hospital Hillerød Denmark; ^3^ Faculty of Health and Medical Sciences University of Copenhagen Copenhagen Denmark; ^4^ Department of Cardiology Herlev and Gentofte University Hospital Herlev Denmark

## Abstract

**Background:**

Caregivers of stroke survivors often face challenges due to the complex nature of caregiving responsibilities and the lack of adequate support systems. Understanding their burden of care and experiences with healthcare professionals is crucial for improving caregiver well‐being and patient outcomes.

**Purpose:**

Was to identify unmet needs for support among caregivers of stroke survivors by exploring their burden of care and experiences of collaboration and support provided by health professionals.

**Method:**

Qualitative semi‐structured interviews were conducted with informal caregivers of stroke survivors. Participants were recruited from a university hospital, among caregivers of patients discharged after a moderate to severe stroke (modified Rankin Scale score 3–5). Thematic analysis was conducted to identify recurring patterns and themes within the data.

**Results:**

Fifteen participants were interviewed, and three interconnected themes were constructed from the data: “New roles and a new life,” “conflicting expectations,” and “unmet needs for information and advice.” Caregivers described the burden of assuming multiple responsibilities, navigating expectations, and adapting their home and life to accommodate the needs of the stroke survivor. Further, they expressed dissatisfaction with the support and information provided by healthcare professionals, highlighting unmet needs for clearer communication, follow‐up and guidance.

**Conclusion:**

Caregivers of stroke survivors experience a significant burden. The findings show poor communication, care continuity, and information to support caregivers. Meeting those needs may reduce caregiver burden and enhance their experience with the healthcare system. This highlights the need for more stroke‐specific education and targeted research on effective interventions.

## 1 Introduction

More than 12 million people globally are affected by stroke each year, making it one of the leading causes of death and disability worldwide [1]. As many as 22%–44% of stroke survivors will be partially or fully dependent on help from others after six months [2]. Stroke not only affects the survivor but also profoundly disrupts the life of the survivor's relatives [3]. An informal caregiver (caregiver), defined [4] as “a person of any age who provides unpaid help and support to a relative, friend, or neighbour who cannot manage to live independently without the carer's help due to frailty, illness, disability or addiction,” plays an important part in the treatment, care, and rehabilitation of stroke survivors and often takes over household chores and caregiving for the stroke survivor ([5]). Caregiving tasks can include managing personal care, monitoring health and illness, administering medication, planning and coordinating social activities, and managing the person's finances, all of which may be roles which the caregiver is unprepared to take on [6]. The trajectory of caring for a stroke survivor often begins suddenly, which explains the general lack of preparation for the role. The many roles can negatively affect caregivers' well‐being [6, 7]. Among negative health effects are a high prevalence of anxiety and depressive symptoms in caregivers. A systematic review reported a pooled estimate of 21% with anxiety symptoms, whereas 40% reported depressive symptoms [8]. Caring for a stroke survivor can be complex and varies considerably based on the cognitive and functional capacities and caregiving needs of the survivor [6].

Informal caregivers of stroke survivors face a wide range of emotional, physical, and social challenges. While caregiving can be associated with positive experiences such as increased self‐esteem, feeling appreciated, and a strengthened relationship with the stroke survivor [9, 10], it can also result in significant caregiver burden, including frustration, fatigue, anxiety, and sadness [2, 9, 11].

Caregiving tasks can include managing personal care, monitoring health and illness, administering medication, planning and coordinating social activities, and managing the person's finances [6]. The trajectory of caring for a stroke survivor often begins suddenly, which explains the general lack of preparation. As a result, caregivers commonly report a lack of support and guidance in adjusting to their new roles. A meta‐synthesis by Quinn et al. found that many spouses felt expected to “just cope,” often without adequate information or support from health professionals [12].

The need for more structured and stroke‐specific support is echoed in an overview by Lou et al. [3], which highlights how caregivers frequently struggle with finding timely and relevant information, particularly regarding what to expect in daily life after stroke [3]. This is further supported by Liljehult et al. [13], who found that even in cases of minor stroke, patients and caregivers experienced a lack of coordinated follow‐up and insufficient knowledge among primary care providers [13].

A recent systematic review titled *Expressing the Burden of Caregivers for Stroke Survivors* emphasises that addressing caregiver burden is essential to stroke care. It stresses the importance of recognising caregiver strain within therapeutic settings and calls for tailored support systems and interventions [14]. Additionally, Garnett and colleagues [15] report that caregivers face considerable difficulties accessing formal health and social services, pointing to broader systemic challenges. These findings are consistent with Denham et al. (2022), who identified emotional support, help with self‐care, and communication with healthcare professionals as among the most unmet needs [9].

Collectively, these studies reveal a gap in understanding how caregivers experience burden over time and how health professionals support them. Despite growing awareness, knowledge remains limited regarding caregivers' perceptions of support and collaboration with the healthcare system post‐stroke.

The purposes of this study are therefore twofold:

1. To identify unmet needs for support among informal caregivers of stroke survivors by exploring their burden of care.
2. To examine how they experience the collaboration and support provided by health professionals.

By addressing this gap, the study aims to contribute knowledge to the development of more targeted, person‐centred support strategies that acknowledge the emotional and relational dimensions of caregiving.

## 2 Methods

### 2.1 Design

The study employed a qualitative descriptive design, grounded in a pragmatic and interpretive epistemology [16]. The approach is inspired by hermeneutic principles, focusing on understanding how caregivers make meaning of their experiences in context. Data were collected through semi‐structured interviews and analysed using thematic analysis [17], allowing for both descriptive depth and interpretive insights.

### 2.2 Participants and Procedures

Participants were recruited from an acute stroke ward at a university hospital in Denmark. All stroke survivors ≥ 18 years admitted to the ward from 1 July 2021 to 30 June 2022 with ischemic or haemorrhagic stroke (ICD‐10 I61, I63, I64) were screened for eligibility by clinical staff in collaboration with the research team. All stroke survivors and their caregivers were included in the study if the survivor met the inclusion criteria of (1) a modified Rankin Scale (mRS) score of 3–5 points at discharge, (2) discharged to their own home or a primary sector rehabilitation facility, and (3) both the survivor and the caregiver gave informed consent (Appendix A, showing mRS). Stroke survivors and caregivers were excluded if the survivor was suffering from a terminal disease, discharged to a nursing home or other hospital ward, or died before they got home.

We included all individuals whom the eligible stroke survivors identified as their informal caregivers and who gave consent to be contacted. This broad definition was chosen to reflect the survivor's own perception of who their caregiver was, which we considered ethically appropriate. While previous studies suggest that the caregiving experience may vary depending on the caregiver's relationship to the patient (e.g., spouse vs. adult child), we deliberately used inclusive criteria to capture a wider range of perspectives on unmet needs. Although the interviews revealed some variation in the type of burden experienced, the key needs and themes were consistent across all participants.

Caregivers were only contacted after the stroke survivor had given consent. Twenty‐seven stroke survivors and their caregivers consented to be contacted for an interview. We aimed to interview the caregiver approximately 1–6 months after the stroke survivor had returned home because they needed time to settle into their new everyday roles. In the end, fifteen caregivers participated.

An interview guide (Appendix B) containing both broad and specific questions was developed through discussions in the interdisciplinary research team and based on research literature [17]. The interview guide was tested by conducting one pilot interview and adjusted based on feedback. Small changes were subsequently made, such as asking more in‐depth about the stroke survivors' level of functioning. All interviews were digitally recorded.

The interviews were conducted by a researcher who was not part of the treatment team (L.W.S). This decision was made to ensure a safe space where caregivers could speak openly about their experiences without concern for how it might affect the care their relative received. The interviewer contacted participants in advance to arrange the interview and began building the relationship during this initial conversation to help establish trust before the interview.

Although the first interview was conducted face‐to‐face, the remaining interviews were held by telephone due to COVID‐19 and time constraints.

### 2.3 Transcription and Analysis

We transcribed all the interviews verbatim. All transcripts were uploaded to NVivo (version 1.7) (QSR International).

The interviews were analysed using a data‐driven thematic analysis as described by Crabtree and Miller [16]. Familiarisation with the data involved listening several times to the recordings and closely reading the transcripts. The transcripts were coded using NVivo. The first and second authors read each interview to gain an overall impression and then performed individual open and descriptive coding of all interviews. After this, their codes were merged in the NVivo collaboration cloud. The codes were compared, and new codes and categories were generated in collaboration between the first and second authors.

**FIGURE 1:**
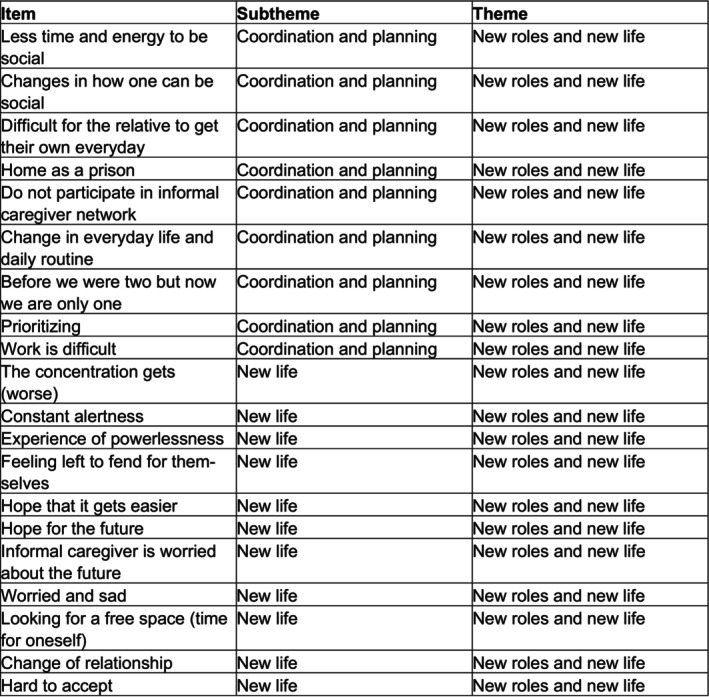
Illustration of the analysis process of Theme 1.

The full interviews, codes, categories, and data were shared with the whole research team. The team met at a workshop, before which all members had read a sample of three interviews and the preliminary codes made by the first and second authors (L.W.S. and M.S.M.). During the workshop, the preliminary codes were re‐coded and re‐categorised, resulting in three themes through researcher triangulation. To enhance and finalise the themes, all the coded text in each theme was reread and discussed by the first and second authors and again with the research team. Figure 1 demonstrates the analysis process of theme 1.

### 2.4 Trustworthiness

To assure the trustworthiness of the findings, the following strategies were applied. One author conducted the interviews, and two authors analysed the data independently of each other. In this way, the research team was able to naively interpret the results without having been present at the interviews. The rest of the research group also interpreted the results and codes. Some of the authors have different clinical and research backgrounds and thus provided new perspectives on the findings. This approach enabled discussions and categorisations that might have been narrower if all authors had had the same background. This approach minimised bias, encouraged broader interpretations, and led to conversations about disparities, resulting in a more comprehensive analysis.

### 2.5 Ethical Considerations

Both stroke survivors and caregivers provided informed consent before inclusion in the study. The study was approved by the local data protection agency (Case No. P‐2021–225). Under Danish legislation, qualitative research is not subject to ethical approval. To ensure the participants' confidentiality, all data were pseudo‐anonymised and handled in accordance with the guidelines of the Danish Data Protection Agency and the European Union's General Data Protection Regulation and complied with the Declaration of Helsinki.

## 3 Results

Fifteen caregivers were interviewed. The interviews lasted from 30 to 90 min. We interviewed 12 women and three men. Nine of the caregivers were spouses or partners, four were children of the stroke survivor, and the remainder had other relationships such as a neighbour, niece, sister, or brother (see Appendix C).

Three overarching but interconnected themes were identified through the data analysis: “New roles and a new life,” “conflicting expectations,” and “unmet needs for information and advice.”

### 3.1 Theme 1: New Roles and a New Life

The caregivers generally stated that the diagnosis changed their lives and relationship to the stroke survivor in many ways.

#### 3.1.1 Sub‐Theme: Coordinating and Planning

Because of the stroke survivor's lack of ability to help in running the household, the caregiver was left with new tasks that they did not necessarily have before the stroke. They were suddenly in charge of cleaning, cooking, gardening, pets, grocery shopping, washing clothes, financial matters, and digital communication and coordination with healthcare professionals, friends, and family. The coordination and planning felt to be a great burden that required considerable time and resources. Some caregivers had to learn new skills, such as cooking. Others described themselves as chauffeurs because the stroke survivor had to be driven to all health‐related appointments. For some, all the planning and coordination changed their relationship with the stroke survivor. Some described it as a great burden with too many tasks, leaving them with no time for themselves. One caregiver described the practical constraints: “I mean, there isn't much time for yourself, you don't have uninterrupted hours or days or anything like that. Just so I could do something else, because everything needs to be considered in terms of how we can leave the house and how long I can leave my husband alone.” (ID 1).

Some found it helpful when the stroke survivor could go to a day care centre, giving them time off for activities that improved their quality of life. However, others had less benefit because taking the stroke survivor to the centre was yet another planning and coordinating task. Some had found the time frame for the visits to be too restricted, and others realised that they could not rely on the time frame because of the varying pickup and drop‐off times of taxis.

Most caregivers stated that everything had to be planned, even a trip to the supermarket. “There isn't much left where it's just ‘easy’, and we just do it. It requires planning and organising. So in that way, it's completely changed our lives.” (ID 4).

In addition, the relationship was affected, especially when the caregiver had to take on a nursing role for the stroke survivor. The caregivers assisted with showers, toilet visits, changing diapers, general hygiene, and intimate tasks.

For some caregivers who were still working, it was difficult to find a work‐life balance. The tasks also resulted in less social activity with friends and family because of exhaustion, while for some, it was a practical problem. One caregiver said: “You suddenly have to see life like a disabled person” (ID 1). Before going to social events, the caregiver had to check if it was safe, if there were stairs, and if there was a handicap‐friendly toilet. In some cases, they had to remember diapers and plan how the stroke survivor could rest.

None of the caregivers had joined networks with other caregivers. Some had not received information from the hospital or family physician about the various possibilities. Some found it impossible to leave home for several hours to participate in a network or consult a psychologist. When asked about such offers, one caregiver said: “No, because how am I supposed to get away?” (ID 2). “I'm in a kind of prison here, you know. We shouldn't make it worse than it is, but obviously our options are extremely limited (ID 2)”.

The caregivers were, in some cases, elderly people with illnesses themselves, and coordination and planning required considerable help from other relatives, networks, and health professionals.

#### 3.1.2 Sub Theme: A New Life

The caregivers’ future hopes and dreams had suddenly changed, especially for those who were partners or spouses. The retirement they had imagined and planned suddenly changed with the stroke.

Some of the caregivers were children of the stroke survivor. The relationship changed when the stroke survivor became dependent on their son or daughter. Children had to take care of their parents while balancing their own lives and emotions. One explained: “But suddenly you become a mother for your own mother, and it's a very reversed relationship, with the extra attention and uncertainty about whether it will happen again.” (ID 5).

Whether they lived together or not, many participants described being constantly alert. One wife living with the stroke survivor described: “I'm also very attentive to, how should I put it, noises, and outbursts. If he's just in the house and I can't see him and he does something, I'll jump up. It's simply because I'm really afraid he'll fall.” (ID 4). A further challenge was to find time to relax and gather one's thoughts. It was difficult for many to have time to do what they wanted to do, such as reading a book. One put it this way: “I mean, in reality [laughs], just an hour to close my eyes and forget how things really are… I hope you understand what I mean. Like sticking my head in the sand, knowing that everything will still be there when I take it out again.” (ID 4).

Hope was described in many ways. The majority hoped that the stroke survivor would regain strength and that it would become easier with time. One caregiver explained: “If the last six months continue and it's going to be like that in the future, umm, then it's maybe just a bit too tough. I mean, we're hoping it'll get better, and then we can do those things again [things that they used to do together]” (ID 8). For others, the stroke survivor was in such a bad state that they were hoping they would pass away: “Uhm, so my greatest wish is that one morning I'll wake up and he's simply passed away, because the life he's living now, it's really not a life.” (ID 6).

This theme illustrates how the stroke profoundly reshaped caregivers' daily lives, roles, and relationships. The transition into caregiving brought a heavy practical burden, often resulting in reduced personal time, social isolation, and emotional strain. Beyond changes in routine, caregiving also affected caregivers' sense of identity and emotional well‐being. Notably, none of the participants had joined support or peer groups, highlighting a lack of accessible services tailored to their needs. Overall, the theme highlights the profound and multifaceted impact caregiving has on a person's lifestyle, identity, and well‐being.

### 3.2 Theme 2: Conflicting Expectations

#### 3.2.1 Sub‐Theme: Rehabilitation or Putting People Away

Several described the time the stroke survivor spent at a temporary rehabilitation centre as demanding. They felt that the person was not receiving the right treatment, but they could not do anything about it. All rehabilitation was organised through the municipality, which differed between municipalities. Most stroke survivors from this study were discharged to a general inpatient rehabilitation centre. These were described as “where they put people away”; other patients had all kinds of illnesses, and there was little knowledge about stroke and no specialised rehabilitation. Many caregivers ended up being frustrated about the treatment and care at the general rehabilitation centre.

“He was in there for almost six weeks, and he just layed in bed, nothing happened. I can assure you that if he'd come home and received home care, he'd be in a better condition today.” (ID 6).

In contrast to the inpatient rehabilitation centres, many caregivers described their collaboration with physiotherapists or occupational therapists as fruitful at home after discharge from the hospital. The stroke survivor benefited from the physical training and exercise, and the caregivers were pleased that it was the same physiotherapist and occupational therapist they saw each time because it created continuity and better collaboration.

#### 3.2.2 Sub‐Theme: Contact With Many Health Professionals Without Continuity

Several caregivers found that the healthcare professionals involved changed rapidly, and many of them provided examples of communication errors and misunderstandings.

“The social worker was replaced, and when I tried to call the municipality, a new person had the case. I think I kind of gave up a bit, and the municipality didn't follow up at all after we came home regarding whether we needed rehabilitation or anything else.” (ID 5).

The conflicting expectations were revealed in communication with the municipality, where some caregivers did not feel heard or listened to: “I don't want a 25‐year‐old guy to wash me… That's what she says… they can't really guarantee that a woman will be the one to come… that can really make me feel frustrated” (ID 9). Another caregiver described cooperation with the municipality in this way: “It damn well needs to improve (laughs)… They need to show up when we make an appointment.” (ID 7).

#### 3.2.3 Sub‐Theme: Turning Down “Help”

The home was described as “a train station” with many different people entering and leaving the house. Some caregivers were disturbed by the help from the municipality. “We handle as much as possible ourselves, also because I don't really want all those people around.*”* (ID 6).

Several had decided to turn down the offer of help from the municipality and provide some of the services themselves because receiving municipal help was more troublesome. One reason was the many different health care professionals coming into the house, which meant that the caregiver had to be there to advise them each time.

*“*New faces constantly appearing, I think that's the most annoying part. You could say if you had two or three people you knew, but there are maybe 10 different ones, no, 15 different ones who come.” (ID 1).

Some of the stroke survivors had aphasia, which made it difficult for them to express their needs to new staff. Instructing health care professionals on how to take care of the stroke survivor was not perceived as a problem in the beginning, but it became demanding over time for the caregiver. It was also described as a burden because of the inflexibility of not knowing at what time of the day the help would be provided. “But she really struggled with having someone come all the time because she found it annoying to have people constantly around, and also, you don't know when they'll show up.” (ID 9).

Caregivers expressed frustration with rehabilitation and support provided by the municipality, citing a lack of stroke‐specific expertise and unmet expectations, while home‐based therapy provided better continuity and collaboration. Frequent staff change, poor communication, and inadequate follow‐up led some to reject municipal help altogether.

### 3.3 Theme 3: Unmet Needs for Information and Advice

Almost all the caregivers mentioned an unmet need for information from health care professionals. This was evident at the hospital right after the stroke, at the rehabilitation centres, during the transition to moving home and when the stroke survivor had been at home for some time without any care or advice from professionals with knowledge about stroke.

#### 3.3.1 Sub‐Theme: Need for a Follow‐Up Consultation

In general, the caregivers would have liked to receive more and better information about the disease and future expectations in relation to the trajectory, both during hospitalisation and as a follow‐up consultation with a neurologist after discharge. One stated: “Something that I and X miss is that there isn't any form of follow‐up consultation at the hospital where you can talk to the doctor about ‘how things are going,’ ‘what to expect,’ or ‘if there's anything else we can do.’ Our family physician does have a broad understanding of many illnesses, but uhm, they don't have the same level of experience with what happens when someone has a stroke.” (ID 15). Many felt that it would have clarified matters more, and they would have felt better supported if they had received a follow‐up consultation. They felt abandoned by the healthcare system, being left with many unanswered questions.

Another caregiver stated, “The ideal scenario would be places where they solely focus on neurology. Where they follow up when you're discharged from the hospital, and where there are people with the right expertise and knowledge…Where there are people who follow up because we have no sense of whether we're falling behind or if she's doing well at her current stage.” (ID 9).

#### 3.3.2 Sub‐Theme: Lack of Information, Communication, and Advice

Some caregivers reported being worried that the survivor would have another stroke. It was difficult for them to get information about the illness and the recovery prognosis from health care professionals. That placed the caregivers under an emotional burden. They felt that they were “left with the burden”, and some had received no support from professionals. “I might have expected to receive, like, a brochure or pamphlet with some information, specifically for caregivers, sort of, key points… But no one mentioned anything… not because I can't look it up myself, but so that you kind of feel supported in this situation you suddenly find yourself thrown into.” (ID 14).

These feelings were expressed in different ways by the caregivers. One of them compared it to a battle where she was fighting on her own without support from healthcare professionals when she was asked how they had supported her in her role as a caregiver: “I've had to figure it out myself and fight my way through it. Well, I don't feel like I've received any support…No one has come to me and asked ‘how are you doing?’ No, they haven't.” (ID 6).

Another caregiver expressed a lack of support and guidance when asked: “How have you felt supported by healthcare professionals in maintaining your everyday life?” A: “Not at all. I don't think that's been a focus at all.” (ID 5).

Some described how they felt forgotten by the system. “So now we haven't had any visits whatsoever. We've also never been contacted by the home care service, such as for cleaning at home. So X wouldn't be able to clean here, and they basically don't know I'm here. So technically, he's listed as single. The municipality has never called and asked about anything.” (ID 15).

Communication at the hospital also lacked information and, therefore, left the caregivers with many unanswered questions in the acute situation. “Well, they were kind, accommodating, and friendly, but there was really no information provided. Information about what had happened, her condition, the future – what will happen in the future? What can we expect from her after two months, after six months, after one year?” (ID 7).

Caregivers consistently reported a lack of timely, clear, and relevant information from hospitalisation to life at home. Overall, the theme highlights the critical need for structured, ongoing communication, and guidance for caregivers.

## 4 Discussion

In this qualitative interview study, we found that being a caregiver for a person with moderate to severe stroke involved a considerable burden related to new roles and everyday tasks, conflicting expectations with health care services, and an unmet need for information and guidance.

The findings of the theme “New Roles and a New Life” highlight the profound and multifaceted impact that caregiving has on daily life. The caregivers in this study described a sudden and often overwhelming shift in roles and responsibilities following the stroke. Ranging from managing household duties to providing intimate personal care, navigating health services, and maintaining constant vigilance. These new demands frequently disrupted their own routines, social lives, and sense of self.

A striking finding was that none of the caregivers had joined any form of support group or caregiver network, with many reporting either a lack of information about such opportunities or practical barriers that made participation impossible. This strongly supports findings from Garnett and colleagues [15], who identified systemic barriers that hinder caregivers' access to formal health and social services. It also aligns with Denham and colleagues [9], who reported that caregivers frequently experience unmet needs for emotional support and guidance, and that support systems are often inaccessible or poorly matched to caregivers' real‐life situations.

The absence of support options is not just a burden to the caregiver; it also has implications for the stroke survivor. As emphasised in a recent systematic review [14], caregiver strain must be recognised within therapeutic settings, as it impacts not only the mental and physical health of the caregiver but also the quality of care provided to the stroke survivor. When caregivers are exhausted, isolated, or unsupported, the risk of burnout increases, reducing their ability to sustain compassionate and effective care.

Moreover, while some caregivers in this study spoke of hope and adaptation, many described feeling trapped, isolated, or emotionally distressed, corresponding with earlier studies [2, 11] that associate caregiving with psychological strain, anxiety, and fatigue. These findings reinforce the urgent need for targeted and sustainable interventions that acknowledge the holistic impact of caregiving.

Overall, this theme illustrates the central argument of this study that informal caregivers often shoulder an invisible burden with little structured support, and that this gap must be addressed through more person‐centred, accessible, and flexible support strategies, both for the sake of the caregiver and the stroke survivor they care for.

The theme “Conflicting Expectations” revealed a mismatch between caregivers' expectations and the support they received, particularly within rehabilitation services and municipal care. Many experienced inpatient rehabilitation as inadequate, lacking stroke‐specific expertise and individualised care, which led caregivers to take on additional responsibilities at home. These findings echo the work of Denham and colleagues [9] and Garnett and colleagues [15], who identified significant structural barriers and unmet needs in accessing appropriate formal services. Caregivers in our study also reported frustration with the inconsistency and unpredictability of home care, leading several to decline municipal support despite feeling overwhelmed. This reflects a broader caregiver burden, as described previously [14], and illustrates how the lack of tailored, reliable services increases both emotional and practical strain. Another study [6] highlights that the sudden and complex nature of caregiving often leaves caregivers unprepared, making continuity, collaboration, and respectful communication from healthcare professionals essential. Without consistent and coordinated support, caregivers are left to navigate fragmented systems that fall short of meeting both their needs and those of the stroke survivor.

The theme “Unmet Needs for Information and Advice” highlights a consistent and significant gap in the support caregivers receive, particularly in relation to information, education, and follow‐up. Caregivers in this study expressed a strong need for follow‐up consultations with neurologists or specialist nurses and for clearer information about the illness, prognosis, and practical aspects of caregiving. This aligns with Denham and colleagues [9], who found that education and specific stroke information were among the most reported long‐term unmet needs. Caregivers not only needed to understand the stroke itself but also how to manage everyday care, recognise warning signs, and adjust to emotional, and lifestyle changes. These findings echo earlier reviews [3, 12], which emphasise that caregivers often struggle to find relevant information and are expected to manage independently without adequate guidance. The theme of “just getting on with it” was reflected in this study, too, where several participants described being left alone with unanswered questions and little contact with professionals after discharge.

The lack of stroke‐specific knowledge in primary care and poor coordination across healthcare sectors may contribute to these gaps, as also suggested by Liljehult and colleagues [13]. Furthermore, the desire for clarity and predictability mirrors the findings of another study [2], where nearly half of caregivers for dependent stroke survivors reported insufficient knowledge about stroke and unmet needs for support.

These findings further support calls for structured follow‐up. The current study supports recent guidelines from the National Institute for Health and Care Excellence in the United Kingdom (NICE UK) recommending a formal review of both patient and caregiver needs six months post‐stroke and annually thereafter [18]. Such reviews could provide crucial touchpoints for emotional support, education, and connection to relevant services, ensuring that caregivers are not left navigating these challenges alone.

We conducted interviews 1–6 months after the stroke survivor's return home to allow caregivers time to adjust to their new roles. This timing enabled rich reflections on their experiences. However, the variation in timing may have influenced responses, with some participants still in transition and others more settled. This diversity is seen as a strength but should be considered when interpreting the findings.

### 4.1 Clinical Implications

Healthcare systems must formally recognise caregivers as key stakeholders in stroke recovery and integrate caregiver support into the care continuum. This includes offering structured follow‐up consultations that involve both stroke survivors and caregivers.

There is also a call for better and timely stroke‐specific information and guidance tailored to the caregiver's situation and capacity. Standardised educational materials and personalised conversations are needed to prepare caregivers for their new roles.

Municipal and rehabilitation services should prioritise continuity of care by minimising turnover among professionals, ensuring predictable support, and fostering long‐term relationships with caregivers.

The absence of caregiver participation in networks or support groups suggests a need to promote and facilitate access to communities. Also, the NICE UK (2023) guidelines should be actively implemented in clinical practice to enhance care and include caregivers of stroke survivors in an adequate way [18].

### 4.2 Education and Research

Healthcare professionals in primary and municipal care require ongoing training in stroke rehabilitation, caregiver communication, and the importance of continuity. Education should be embedded in post‐stroke care, not treated as a one‐time intervention. Additionally, further research is needed to develop and evaluate tailored, scalable interventions for caregivers, explore how their needs change over time, and assess how national guidelines can be effectively implemented in practice.

### 4.3 Limitations

This study has several limitations that we wish to acknowledge. All interviews, but one, were conducted by phone due to the COVID‐19 pandemic, which may have affected rapport and participants' experiences, given the altered healthcare context. The same interviewer conducted all interviews and was not involved in participants' care, which may have influenced the dynamic but was intended to encourage open and critical reflection. The interviewer's clinical background as a nurse helped support communication in sensitive situations.

We used a convenience sample focused on caregivers of stroke survivors with significant impairment. Some survivors could not consent, and a few caregivers declined due to high burden. We did manage to interview caregivers who had different backgrounds: Some were in active employment, some were retired, and some lived with stroke survivors. Most participants were spouses, and more were female than male, which may limit the diversity of perspectives. The stroke survivors selected which caregivers we could contact. Being a caregiver is a complex situation, and different caregivers take on varying roles. Some perspectives and aspects of the caregiver role might not be represented sufficiently in the study.

The interviews were conducted approximately 1–6 months after the stroke survivor returned home and may thus have been influenced by recall bias for some, and the time frame may have affected the experience of being an informal caregiver. The interpretation of qualitative data is subjective, and the findings may have been influenced by the researcher's perspectives or preconceptions. The analysis of each interview may be limited, potentially overlooking nuances or important themes within the data, although we used researcher triangulation to limit that bias, where two independent researchers analysed the data, and the research group came together to form and discuss the final themes. All researchers had different backgrounds and introduced different perspectives into the analysis. Although thematic saturation was approached, we acknowledge that the relatively small sample size and variation in the timing of interviews (1–6 months post‐discharge) may have limited the full range of caregiver experiences. This may have influenced the depth and diversity of the themes identified. This study focuses on a specific context and population, and the findings may not be directly transferable to other settings or caregiver groups.

## 5 Conclusion

This qualitative study confirmed the significant burden and unmet needs of informal caregivers of stroke survivors. Caregivers experience profound changes in their roles and daily lives, often facing conflicting expectations and inadequate support from healthcare professionals. The findings highlight the necessity for improved communication, continuity of care, and comprehensive information to support caregivers effectively. Integrating caregivers into the care plan and providing tailored rehabilitation services may enhance both caregiver and patient outcomes. Addressing these needs might alleviate some of the burden and enhance stroke survivors' and their caregivers' experience of being supported by the healthcare system.

## Author Contributions

D.O. and J.L. designed the study. L.W.S. conducted the interviews. L.W.S. and M.S.M. analysed the interviews with analysis triangulation by L.W.S., M.S.M., J.L., M.M.L. and S.S.R. L.W.S., M.S.M. and S.S.R. wrote the first draft, and J.L, M.M.L. and D.O. contributed substantially to its revision. All authors participated in reviewing and rewriting the manuscript and approved the final version for publication.

## Funding

This work was supported by the Novo Nordisk Fonden (0059016).

## Conflicts of Interest

The authors declare no conflicts of interest.

## Data Availability

The data that support the findings of this study are available from the corresponding author upon reasonable request.

## Appendix A

Modified ranking scale.

The figure is from: Brain injury after cardiac arrest: Pathophysiology, treatment, and prognosis (2021).

## Appendix B

Interview guide.

**Table:**

Research question	Interview questions
Presentation of myself; *p*resentation of the purpose of the study; formalities regarding the interview—breaks, anonymity, etc.; signed consent form	
From the caregiver's perspective, what types of support do patients need and who besides caregivers helps the patient in the first six months after stroke?	What is your relationship to the patient? What is the physical functioning level of the patient? How long have you been supporting the patient in their own home? Mention other relatives who support the patient. (Complete the relationship card) Who has helped you recently? What support do the other relatives offer to the patient? How much time do you think the different relatives spend on tasks for or with the patient? How challenging do you think the different relatives find the support they offer to the patient? How do you find the support offered by the different relatives to the patient?
How do caregivers feel about the support offered to the patient?	What is your experience of your role as a relative? Do you feel that your help is sufficient? Do you feel that you have time and energy to meet the patient's needs? Who supports you? How are things in your family? How do you handle things in your family? Are there any challenges related to your workplace?
How do caregivers find the support that healthcare professionals offer the patient?	How do you feel healthcare professionals are meeting the patient's needs? How do you find communication with healthcare professionals? What tasks do healthcare professionals assist with? What treatment options have you been offered in relation to addressing the various needs (e.g., physiotherapy, speech therapy, etc.)? How do you perceive the collaboration between yourself and the healthcare professionals in addressing the different needs? Are there any needs of the patient that the healthcare professionals are not aware of? Describe what typical communication with healthcare professionals entails. Whom do you contact? (the same person, multiple individuals, etc.) How do you communicate? (phone, email, FaceTime, etc.)
How do caregivers find the support that healthcare professionals offer them as informal caregivers?	How have you felt supported by healthcare professionals in maintaining your everyday life? How do you feel about the guidance and instruction provided by healthcare professionals to help you support the patient? How can healthcare professionals support you in your role as a caregiver? What concerns do you have about your own situation? What is your experience of healthcare professionals inquiring about you and how you are feeling?
Follow‐up questions	Has anyone helped you and the patient in the last 1–2 months that we haven't mentioned? If you could get all the support you need, what would it be? And from whom? Why is this relationship special?

## Appendix C

Data table with participant information.

**Table:**

Record ID	Relation to patient	Gender	Age	Admitted to	Patient gender	Patients’ approximate age when discharged	Modified Ranked Scale (during admission)	Modified Ranked Scale (habitually)
1	Spouse	Female	> 60	TRC	Male	70–80	4	0
2	Spouse	Female	< 60	Other	Male	60–70	5	0
3	Spouse	Male	> 60	Own home	Female	70–80	4	4
4	Spouse	Female	> 60	TRC	Male	70–80	4	0
5	Child of the patient	Female	< 60	TRC	Female	60–70	4	1
6	Spouse	Female	> 60	TRC	Male	70–80	5	5
7	Spouse	Male	> 60	TRC	Female	70–80	5	0
8	Spouse	Female	> 60	TRC	Male	80–90	4	2
9	Other	Female	> 60	TRC	Female	70–80	4	0
10	Other	Female	< 60	TRC	Female	70–80	4	0
11	Child of the patient	Male	< 60	TRC	Male	70–80	4	1
12	Child of the patient	Female	< 60	TRC	Male	80–90	4	0
13	Spouse	Female	< 60	Own home	Male	60–70	3	0
14	Child of the patient	Female	< 60	TRC	Male	70–80	4	0
15	Partner	Female	> 60	Own home	Male	80–90	3	0

Abbreviation: TRC: Temporary rehabilitation centre.
